# Dystromirs as Serum Biomarkers for Monitoring the Disease Severity in Duchenne Muscular Dystrophy

**DOI:** 10.1371/journal.pone.0080263

**Published:** 2013-11-25

**Authors:** Irina T. Zaharieva, Mattia Calissano, Mariacristina Scoto, Mark Preston, Sebahattin Cirak, Lucy Feng, James Collins, Ryszard Kole, Michela Guglieri, Volker Straub, Kate Bushby, Alessandra Ferlini, Jennifer E. Morgan, Francesco Muntoni

**Affiliations:** 1 The Dubowitz Neuromuscular Centre, Institute of Child Health, University College London, London, United Kingdom; 2 Department of Pathogen Molecular Biology, London School of Hygiene and Tropical Medicine, London, United Kingdom; 3 Childrens National Medical Centre, Research Centre for Genetic Medicine, Washington DC, United States of America; 4 Department of Pediatric Neurology, Cincinnati Children’s Hospital Medical Center, Cincinnati, Ohio, United States of America; 5 Sarepta Therapeutics, Cambridge, Massachusetts, United States of America; 6 Institute of Human Genetics, Newcastle University, Newcastle, United Kingdom; 7 Department of Medical Science, University of Ferrara, Ferrara, Italy; Institut de Myologie, France

## Abstract

Duchenne muscular Dystrophy (DMD) is an inherited disease caused by mutations in the dystrophin gene that disrupt the open reading frame, while in frame mutations result in Becker muscular dystrophy (BMD). Ullrich congenital muscular dystrophy (UCMD) is due to mutations affecting collagen VI genes. Specific muscle miRNAs (dystromirs) are potential non-invasive biomarkers for monitoring the outcome of therapeutic interventions and disease progression. We quantified miR-1, miR-133a,b, miR-206 and miR-31 in serum from patients with DMD, BMD, UCMD and healthy controls. MiR-1, miR-133a,b and miR-206 were upregulated in DMD, but unchanged in UCMD compared to controls. Milder DMD patients had higher levels of dystromirs than more severely affected patients. Patients with low forced vital capacity (FVC) values, indicating respiratory muscle weakness, had low levels of serum miR-1 and miR-133b. There was no significant difference in the level of the dystromirs in BMD compared to controls.

We also assessed the effect of dystrophin restoration on the expression of the five dystromirs in serum of DMD patients treated systemically for 12 weeks with antisense oligomer eteplirsen that induces skipping of exon 51 in the dystrophin gene. The dystromirs were also analysed in muscle biopsies of DMD patients included in a single dose intramuscular eteplirsen clinical trial. Our analysis detected a trend towards normalization of these miRNA between the pre- and post-treatment samples of the systemic trial, which however failed to reach statistical significance. This could possibly be due to the small number of patients and the short duration of these clinical trials.

Although longer term studies are needed to clarify the relationship between dystrophin restoration following therapeutic intervention and the level of circulating miRNAs, our results indicate that miR-1 and miR-133 can be considered as exploratory biomarkers for monitoring the progression of muscle weakness and indirectly the remaining muscle mass in DMD.

## Introduction

Muscular dystrophies are a group of inherited conditions characterised by progressive muscle wasting and weakness with variable severity. The most common muscular dystrophies are dystrophinopathies, caused by mutations in the dystrophin gene that depending on the type of mutation, lead to the severe Duchenne or the milder Becker muscular dystrophy. DMD is an X-linked disorder and recent figures have refined its incidence to 1 in 5,000 live male births [1], [2]. In BMD the clinical course is milder with a later age of onset and its prevalence is ∼ 1:18,450 [3].

Although there is variability in the severity of the disease in individual DMD patients, the clinical course follows a well-described progression. The absence of the dystrophin protein in DMD leads to disruption of the link between the cytoskeleton and the extracellular matrix in the muscle fibres and results in muscle wasting, cycles of muscle fibre regeneration and degeneration, inflammation and gradual replacement of the muscles by connective and adipose tissue. This process is reflected clinically in progressive muscle weakness leading to loss of ambulation by the age of 12 years, and respiratory, cardiac and orthopaedic complications in the second decade of life, leading to premature death [4].

The highly heterogeneous group of congenital muscular dystrophies (CMD) includes a broad range of myopathies, classified in several groups based on the phenotype and the affected gene. There is an increasing number of CMD disease causing genes; among these are collagen VI gene mutations that cause Ullrich congenital muscular dystrophy and the milder Bethlem myopathy allelic variant. Both forms are due to either dominant or recessive mutations in one of *COL6A* genes (*COL6A1*, *COL6A2* and *COL6A3*). In these conditions, the deficiency of collagen VI at the basal membrane leads to disruption of the link of the muscle fibres with the extracellular matrix [5]. While in both DMD and UCMD the primary genetic defect results in myofiber wasting and replacement by fibro-adipose tissue, the two disorders have major differences in their pathogenesis and disease manifestations.

Micro RNAs (miRNA) are small RNA sequences (∼22 nucleotides) that act as post-transcriptional regulators through binding to the target mRNA causing repression of the target gene by inducing mRNA degradation or translational inhibition. Micro RNAs are conserved in different organisms suggesting a vital role of these small RNA molecules in regulation of wide range of biological processes such as development, differentiation, proliferation and cell death. Aberrant miRNA expression has been shown in different diseases, e.g. cancer, heart disease, myocardial infarction and also in muscular dystrophies [6], [7], [8]. MiR-1, miR-133a, miR-133b, miR-31 and miR-206 have been referred as dystromirs in previous studies due to their specific expression in muscle cells and their role in skeletal muscle maintenance and regeneration [9]. The expression of miR-1 is initiated by myogenic regulatory factors and promotes terminal differentiation. Although miR-1 and miR-133a are expressed from the same transcript, they have opposite functions in skeletal muscle, with miR-133a enhancing myoblast proliferation [10]. MiR-206 and miR-133b are also encoded by a single noncoding RNA expressed only in skeletal muscles [11]. MiR-206 is expressed in satellite cells, quiescent muscle precursor cells that contribute to muscle regeneration [12] and promotes their differentiation and fusion into multinucleated myotubes [13]. MiR-31 modulates dystrophin expression by targeting the 3’UTR of dystrophin transcript and thus repressing the translation of dystrophin mRNA [14]. MiR-31 has been suggested as suitable target in ameliorating strategies for enhancing dystrophin restoration in exon skipping DMD therapy [14].

Several studies shed light on the role of microRNAs in the pathological pathways activated in dystrophic skeletal muscle [6], [9], [14], [15]. Cacchiarelli and colleagues [9] analysed muscle specific miRNAs in serum samples from 10 DMD patients and found increased levels of miR-1, miR-133 and miR-206 in serum that broadly correlated with the severity of the disease. In a following study, the same miRNAs were also found increased in the serum of the canine X-linked muscular dystrophy model (CXMDJ) over those from age-matched normal dogs [16]. A more complex picture of the expression of miRNAs was revealed in a study carried out by Roberts and colleagues who analysed miRNA expression in different muscles of the *mdx* mouse, a mouse model of DMD [17]. A muscle specific pattern of expression was identified for several miRNAs together with miRNAs showing changes in the same direction between different muscles (miR-31, miR-1, miR-133, miR-206). Interestingly, the level of the dystromirs showed a significant difference between serum and muscle samples. MiR-1, miR-133a and miR-206 were significantly increased in *mdx* serum but downregulated or modestly upregulated in muscle [17].

The studies carried out by Cacchiarelli et al. [9] and Roberts et al. [17] also showed that the exon skipping therapies aimed at dystrophin restoration influence the level of circulating dystromirs in serum of *mdx* mice. In both studies the elevated levels of miR-1 and miR-206 in the serum of *mdx* mice were decreased close to the level in wild type mice following exon skipping therapy and dystrophin restoration in *mdx* mice.

No up-to-date studies have investigated serum circulating miRNAs in UCMD patients and such work is of a particular interest. The marked fibrosis and reduced regeneration potential is a feature of UCMD that would probably reflect the miRNAs and their level in serum.

The present study aims at establishing a baseline of the level of circulating serum miR-1, miR-133a, miR-133b, miR-206 and miR-31 in DMD patients, as the previous studies performed included only a small number of patients. In addition, we aimed to correlate the amount of the selected serum miRNAs to the disease severity in DMD patients, assessed using several parameters such as ambulation status, validated functional assessments (the North Star Ambulatory Assessment score; (NSAA)), cardiomyopathy, presence of severe scoliosis requiring surgery and respiratory forced vital capacity values (FVC). Furthermore, we assessed the effect that intramuscularly or systemically administered antisense morpholino oligomer (eteplirsen), which induces targeted skipping of exon 51 and dystrophin restoration, had on the expression level of these miRNAs in DMD patients included in two completed eteplirsen clinical trials [18], [19]. Lastly, we have compared the levels of circulating serum miR-1, miR-133a, miR-133b, miR-206 and miR-31 found in DMD with those detected in UCMD patients, a muscular dystrophy with completely different pathophysiology compared to DMD.

## Results

### Serum levels of miR-1, miR-206, miR-31, miR-133a and miR-133b in DMD

**Figure 1:**
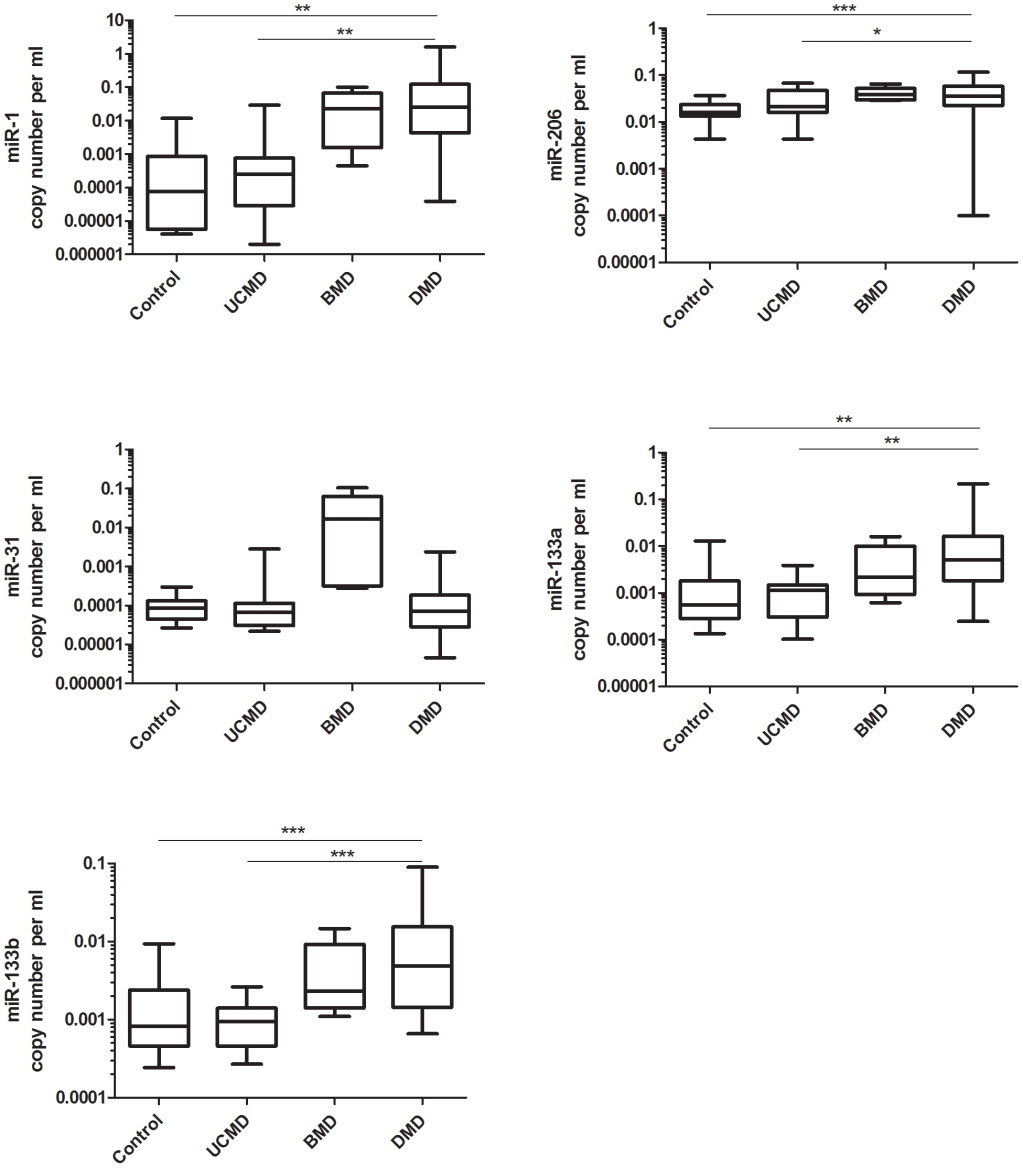
Serum levels of the analysed dystromirs in DMD, BMD, UCMD and controls. Absolute quantification of miR-1, miR-206, miR-31, miR-133a and miR-133b in serum samples of 44 DMD, 5 BMD, 16 UCMD patients and 14 healthy controls. The data are presented in a logarithmic scale as miRNA copy number per ml normalized to the spiked-in *C. elegance* miRNAs (*cel-miR-54, cel-miR-39, cel-miR-238)*. P-values derived from t-test are presented with *, **, *** and correspond to p <0.05, p<0.01 and p<0.001 respectively.

Upregulation of miR-1, miR-133a and miR-206 in serum has been previously reported in a small cohort of DMD patients [9], [20]. Here, with the aim to establish a baseline of the level of serum miR-1, miR-206, miR-31, miR-133a and miR-133b, we quantified the five dystromirs in a large cohort of DMD patients (n = 44) and in BMD patients (n = 5). The copy number of the dystromirs in DMD and BMD was compared to their levels detected in the serum of 14 healthy controls. T-test for unequal means was used to examine the differences in the copy number of the five analysed dystromirs in DMD, BMD patients and controls. Elevated levels in the serum of DMD patients compared to the levels in healthy controls were detected for miR-1 (p = 0.005; 88.7-fold), miR-206 (p<0.0001; 2.35-fold), miR-133a (p = 0.008; 10.17-fold) and miR-133b (p = 0.0008; 6.9-fold) (Figure 1). MiR-31 showed a higher level in DMD compared to healthy controls but the result did not remain significant after multiple comparison correction. MiR-206 showed an increased level in BMD compared to controls (p<0.05) and different level of miR-133a between DMD and BMD patients (p<0.05) was detected but they did not remain significant after multiple testing correction.

### miRNAs as serum biomarkers reflecting disease severity in DMD

Circulating miR-1, miR-133 and miR-206 have been proposed as potential biomarkers for DMD [9], [16], [17]. To study whether miR-1, miR-206, miR-31, miR-133a and miR-133b can serve as non-invasive biomarkers and aid the monitoring of the disease progression, we analysed their levels in the serum of 28 ambulant and 16 non-ambulant DMD patients according to several clinical variables: ambulation status, NSAA, cardiomyopathy, scoliosis severe enough to require surgery, FVC values and steroid regimen at the time when the serum sample was taken. BMD patients were not considered in the analysis, as four out of five BMD patients included in this study were ambulant, without cardiac abnormality and without severe scoliosis.

**Figure 2:**
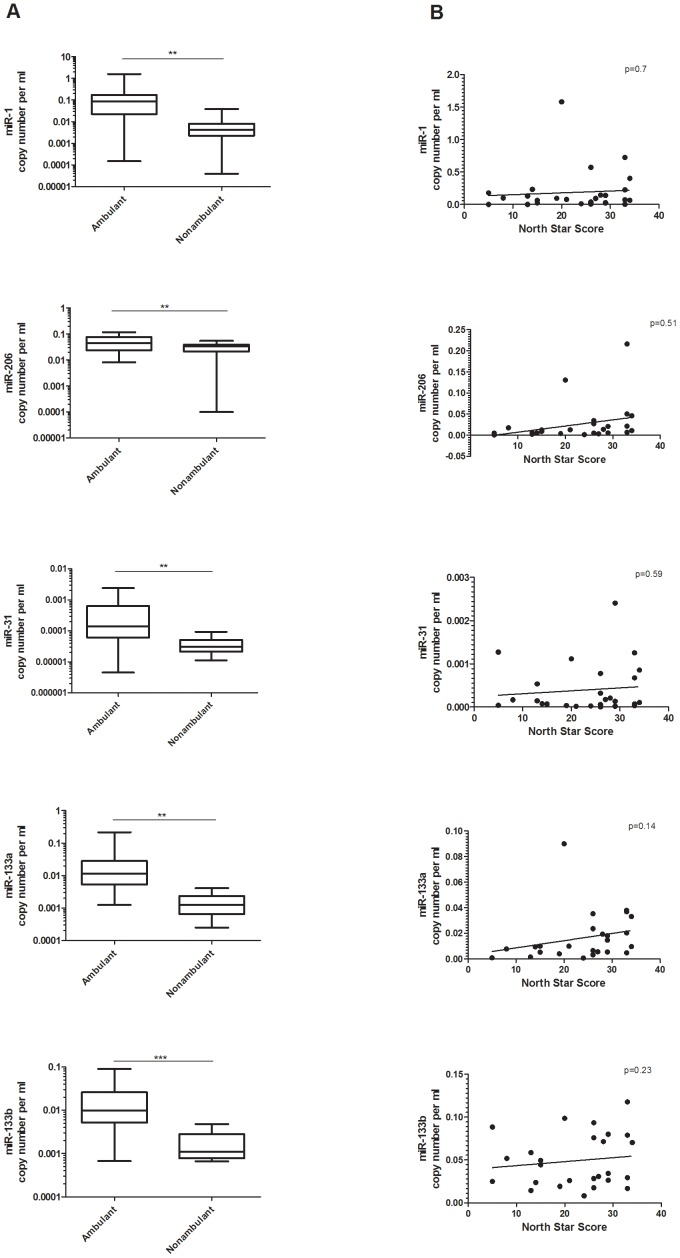
Serum levels of the analysed dystromirs and functional ability of DMD patients. A. MiR-1, miR-206, miR-31, miR-133a and miR-133b in ambulant and non-ambulant DMD patients. B. Correlation analysis between the level of the dystromirs and the NSAA scores. The data are presented in a logarithmic scale as miRNA copy number per ml normalized to the spiked-in *C. elegance* miRNAs (*cel-miR-54, cel-miR-39, cel-miR-238)*. P-values derived from t-test are presented with **, *** and correspond to p<0.01 and p<0.001 respectively.

A significant difference in the copy number for all of the dystromirs was detected, with ambulant patients having higher level of dystromirs compared to non-ambulant patients (Figure 2A). To analyse whether dystromirs quantity in serum could be utilised as biomarker for physical ability in ambulant DMD patients, we performed correlation analysis between the NSAA and the level of the dystromirs in the 26 ambulant DMD patients (age range between 4 and 13 years) with available NSAA score. The analysis showed no correlation between the NSAA score and the amount of dystromirs in serum (Figure 2B).

**Figure 3:**
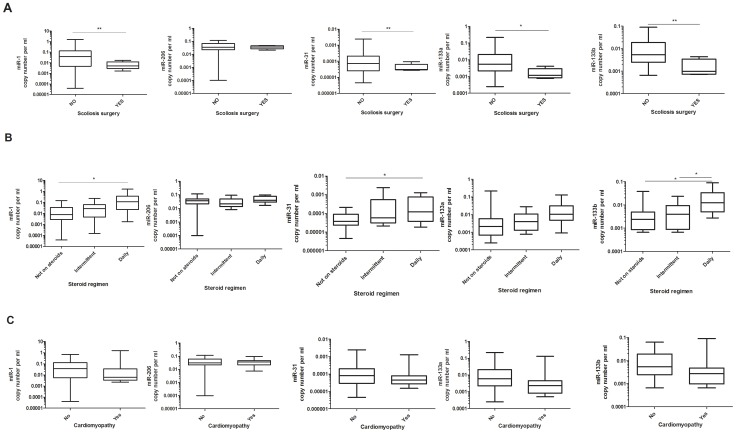
Serum level of the analysed dystromirs in DMD patients according to clinical variables. Level of miR-1, miR-206, miR-31, miR-133a and miR-133b in serum samples of DMD patients: A. without the need of scoliosis surgery and with scoliosis surgery performed or recommended; B. not on glucocorticoid treatment, on intermittent or on daily steroid regimen; C. without cardiomyopathy and with cardiac abnormality. The data are presented in a logarithmic scale as miRNA copy number per ml normalized to the spiked-in *C. elegance* miRNAs (*cel-miR-54, cel-miR-39, cel-miR-238)*. P-values derived from t-test are presented with *, ** and correspond to p <0.05 and p<0.01 respectively.

Scoliosis is a common complication in DMD that often requires surgical intervention [21]. Analysis of the level of the five studied dystromirs showed that patients who either had, or were being considered for, spinal surgery had lower levels of miR-1 (p = 0.0087), miR-31 (p = 0.0025), miR-133a (p = 0.011) and miR-133b (p = 0.0012) compared to patients who had only mild or no scoliosis (Figure 3A). The mean age of the patients who either had, or were considered for, surgery was 15 years and the mean age of the patients with mild or no scoliosis was 10 years. However, the difference in the level of miR-1, miR-31, miR-133a and miR-133b between the two groups was not driven by the age difference as no significant relationship between miRNA copy number and age was detected.

Glucocorticoid treatment is the only intervention that can prolong ambulation, delay respiratory insufficiency and reduce scoliosis in DMD patients [22]. The two commonly used regimens are daily and intermittent (10 days on, 10 days off) glucocorticoid administration. To examine whether there is a difference in the serum level of the five dystromirs, we compared untreated DMD patients with those on either an intermittent or daily regimen (Figure 3B). The analysis showed that patients on the daily, but not the intermittent, regimen had increased copy number of miR-1 (p = 0.036), miR-31 (p = 0.022) and miR-133b (p = 0.041) compared to untreated patients. MiR-133b also showed higher copy number in patients on daily compared to patients on intermittent regimen (p = 0.04). Higher levels of miR-1 (p = 0.025), miR-206 (p =  0.009), miR-133a and miR-133b were detected in patients who were not on glucocorticoid treatment compared to healthy controls indicating that the upregulation of miRNAs in the serum is not due to the glucocorticoid treatment but is a result of the pathological process in DMD patients (Figure S1), as previously demonstrated in *mdx* mice and canine X-linked muscular dystrophy model (CXMDJ) [16], [17], [20].

The dystrophin deficiency in DMD patients also leads to progressive cardiomyopathy with clinically apparent symptoms in 90% of the patients by the age of 18 years [23]. Here, we analysed the level of miR-1, miR-133a,b, miR-206 and miR-31 in DMD patients without clinically manifesting cardiomyopathy (n = 31) and with symptomatic cardiomyopathy (n = 12). None of the analysed dystromirs showed significant differences in their level in patients without cardiac abnormality compared to DMD patients with cardiomyopathy (Figure 3C).

**Figure 4:**
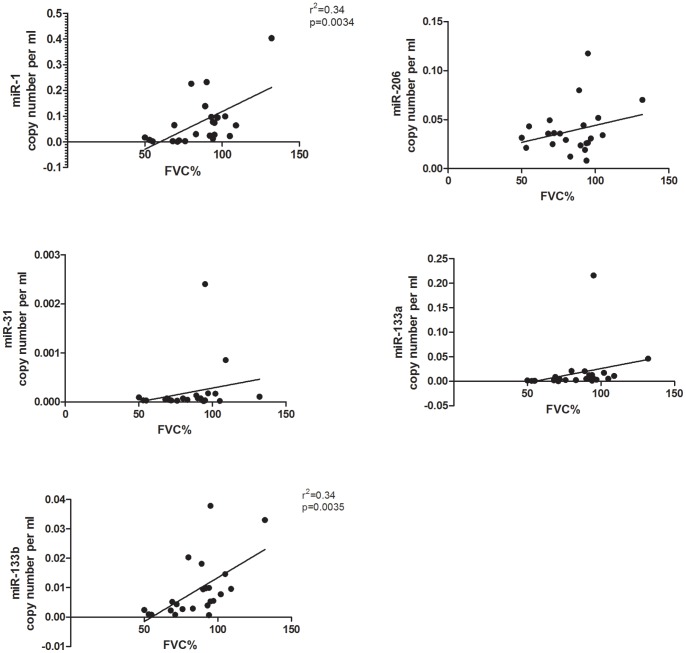
Correlation analysis between the level of the analysed dystromirs in DMD and the FVC values. Linear regression analysis between the levels of miR-1, miR-206, miR-31, miR-133a and miR-133b in serum samples and FVC scores in DMD patients showed correlation between the level of miR-1 (p = 0.0034, r^2^ = 0.34) and miR-133b (p = 0.0035, r^2^ = 0.34) with the FVC scores. The data are presented as miRNA copy number per ml normalized to the spiked-in *C. elegance* miRNA s (*cel-miR-54, cel-miR-39, cel-miR-238*). Regression lines are also presented.

Reduced respiratory function secondary to muscle weakness is present in DMD patients after the first decade of life and respiratory complications are the main cause of mortality. FVC value is an index of respiratory function and can be used as prognostic marker in DMD [24]. Linear regression analysis in all DMD patients (n = 44) was carried out in order to determine whether there is a correlation between the FVC values and the level of dystromirs detected in serum. A positive correlation was observed for miR-1 (r^2^ = 0.34; p = 0.0034) and miR-133b (r^2^ = 0.34; p = 0.0035), indicating that low level of miR-1 and miR-133b are associated with low FVC score (Figure 4).

**Figure 5:**
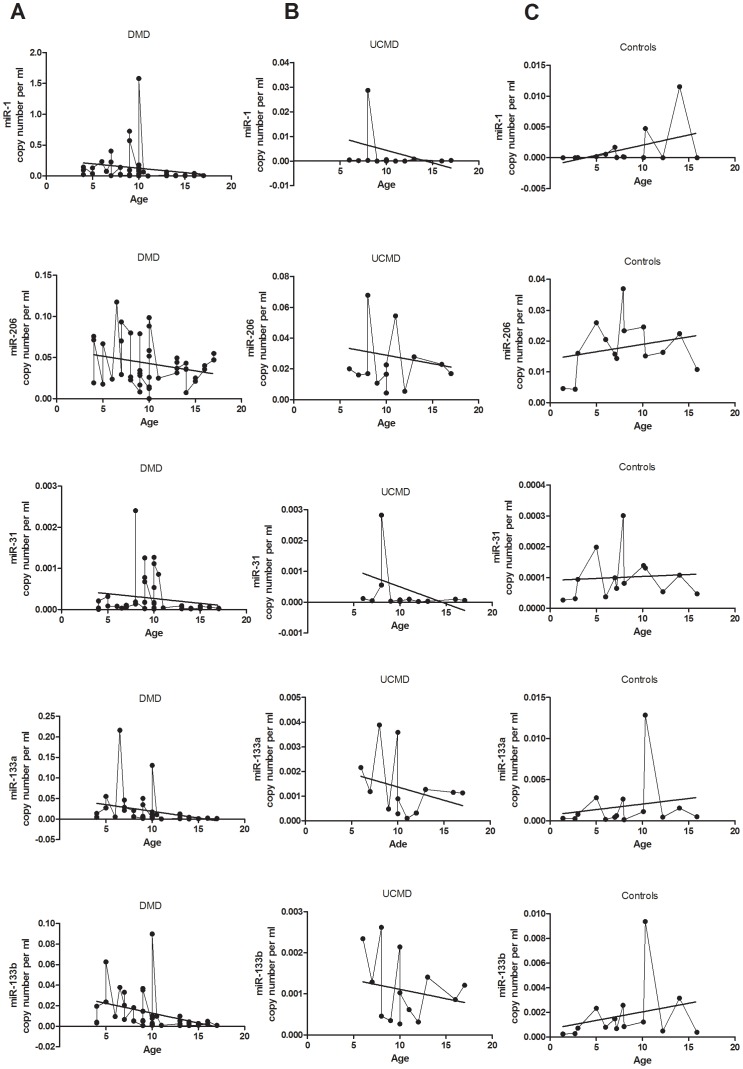
Age profile of miR-1, miR-206, miR-31, miR-133a and miR-133b in serum samples. Level of miR-1, miR-206, miR-31, miR-133a and miR-133b in serum samples according to the age of the: A. DMD patients; B. UCMD patients; C. healthy controls. The data are presented as miRNA copy number per ml normalized to the spiked-in *C. elegance* miRNAs (*cel-miR-54, cel-miR-39, cel-miR-238)*. Regression line is also presented.

In addition, linear regression analysis between the quantity of the dystromirs in serum and the age of DMD patients showed a weak association for miR-133a (p = 0.039) and miR-133b (p = 0.016) but it did not remain statistically significant after Bonferroni correction (Figure 5A). Fluctuating levels of both of these dystromirs were seen, with a slow decrease in patients between 4 to 10 years, followed by a considerable decrease in the amount of miR-133a and miR-133b after the age of 11 years. MiR-1 showed a similar profile; patients between the age of 4 and 10 years had higher levels than patients after the age of 11 years. Increased levels of miR-31 in patients in the age range 8 to 11 years were detected followed again with lower amounts of miR-31 in older patients (12 to 17 years). Fluctuating levels of miR-206 throughout the analysed age range were detected. Decreasing levels of the miRNAs with age were also detected in serum of UCMD patients (Figure 5B), but we did not see a considerable decrease in the amount of miR-133a, miR-133b and miR-1 in older patients, in contrast to our findings in DMD patients. The serum levels of the five dystromirs in healthy controls followed the opposite trend to that found in DMD and UCMD, with a slight increase in the amount of dystromirs with age (Figure 5C).

### miRNAs as biomarkers for the outcome of therapeutic interventions

**Systemic phosphorodiamidate morpholino oligomer (Eteplirsen, AVI-4658) treatment trial.** To elucidate whether the exon skipping therapy and the dystrophin restoration induced by eteplirsen influenced the serum level of the dystromirs, we quantified miR-1, miR-206, miR-31, miR-133a and miR-133b in serum samples taken at baseline (pre-treatment samples) and at week 12 (post-treatment samples) in 12 DMD patients included in the systemic antisense oligomer eteplirsen phase II clinical trial [19]. Eteplirsen induced skipping of exon 51 and restoration of dystrophin protein expression in a dose dependent, but variable, manner in the patients from cohort 3 to cohort 6 (Table 1). Five of the DMD patients (cohort 3, 4, 5, 6) showed a low response, with skipping of exon 51 detected but no detectable increase in dystrophin protein production in the post-treatment muscle biopsy (low responders). Four DMD patients (cohort 5, 6) showed response at RNA level with increase of dystrophin production in post-treatment muscle (responders) and three DMD patients (cohort 3, 5, 6) showed response at RNA level and a greater increase in dystrophin protein production measured with three methods of quantification (good responders) [19].

**Table 1:** Deletion, dose escalating scheme and response of the patients who participated in the systemic eteplirsen clinical trial.

Patient ID	Response to eteplirsen	Cohort	Deletion
P7	+++	Cohort 3 (2 mg/kg)	49–50
P8	+	Cohort 3 (2 mg/kg)	49–50
P10	+	Cohort 4 (4 mg/kg)	48–50
P11	+	Cohort 4 (4 mg/kg)	45–50
P12	++	Cohort 5 (10 mg/kg)	49–50
P13	++	Cohort 5 (10 mg/kg)	48–50
P14	+	Cohort 5 (10 mg/kg)	47–50
P15	+++	Cohort 5 (10 mg/kg)	49–50
P16	+	Cohort 6 (20 mg/kg)	45–50
P17	++	Cohort 6 (20 mg/kg)	45–50
P18	+++	Cohort 6 (20 mg/kg)	49–50
P19	++	Cohort 6 (20 mg/kg)	45–50

Response to eteplirsen: + shows response only at RNA level ; ++ shows response at RNA level and increase of dystrophin expression in post-treatment muscle; +++ shows response at RNA level and a greater increase in dystrophin production in post-treatment muscle measured with three methods of quantification.

**Figure 6:**
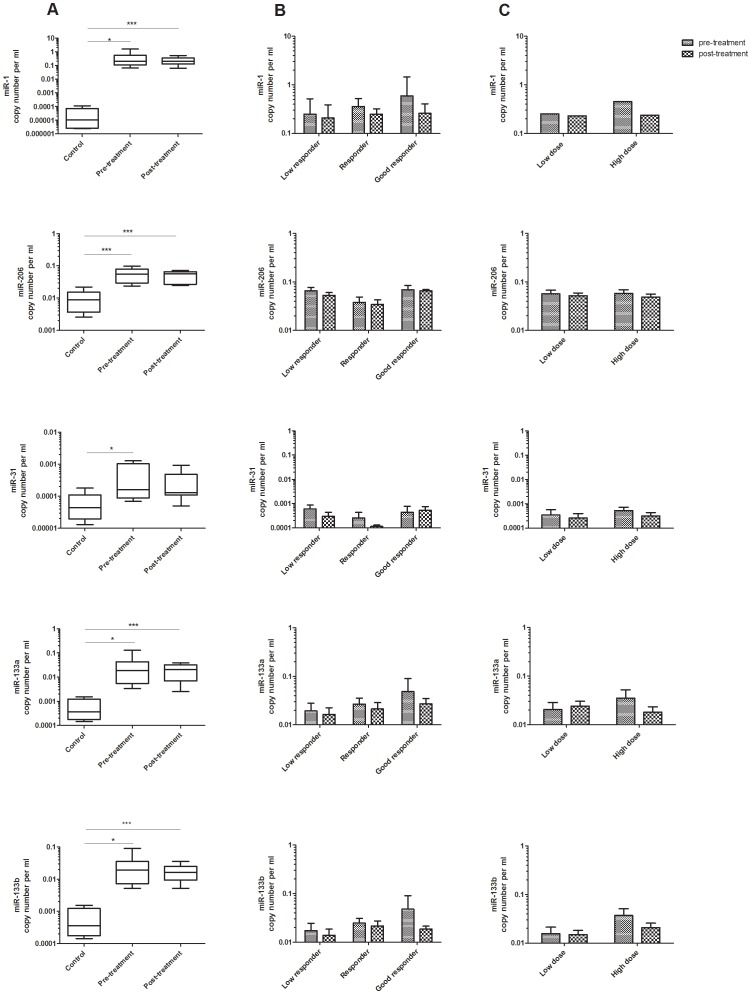
miRNAs as biomarkers for the outcome of therapeutic interventions: Systemic eteplirsen treatment trial. Absolute quantification of miR-1, miR-206, miR-31, miR-133a and miR-133b in serum of: A. DMD patients before treatment with eteplirsen (pre-treatment), after treatment (post-treatment) and controls; B. low responders, responders and good responders; C. patients who received low dose and patients with high dose of eteplirsen. The data are presented in a logarithmic scale as miRNA copy number per ml normalized to the spiked-in *C. elegance* miRNAs (*cel-miR-54, cel-miR-39, cel-miR-238*). P-values derived from two-tailed t-test are presented with *, *** and correspond to p <0.05 and p<0.001 respectively.

An increase in the serum levels of miR-1, miR-206, miR-31, miR-133a and miR-133b in pre-treatment samples compared to controls (n = 14) and an increase of miR-1, miR-206, miR-133a and miR-133b in post-treatment compared to control samples was detected (Figure 6A). We tested the difference in the miRNA levels in pre-treatment and post-treatment samples in all of the analysed patients. We also analysed the correlation between the amount of the selected dystromirs in serum and the response to eteplirsen. The difference between the pre- and post-treatment levels of miR-1, miR-206, miR-31, miR-133a and miR-133b was also tested in the patients who received lower dose (cohort 3 and 4) and in patients who received higher dose of eteplirsen (cohort 5 and 6). Because of the small number of patients included in the trial and the variable patient response, the data analysis did not reveal a conclusive correlation between the amount of miRNAs and the dystrophin levels in patients treated systemically with eteplirsen (Figure 6B and 6C). Analysis of samples from ongoing longer duration studies may better address this issue.

**Intramuscular phosphorodiamidate morpholino oligomer (Eteplirsen, AVI-4658) treatment trial.** Next, we analysed whether the intramuscular administration of eteplirsen influenced the expression of the selected dystromirs in the DMD patients included in the intramuscular eteplirsen clinical trial [18]. A dose-dependent exon skipping was observed and the higher dose of eteplirsen resulted in increased dystrophin expression in treated EDB muscles. No dystrophin protein was detected in the patients who received the low dose of eteplirsen.

**Figure 7:**
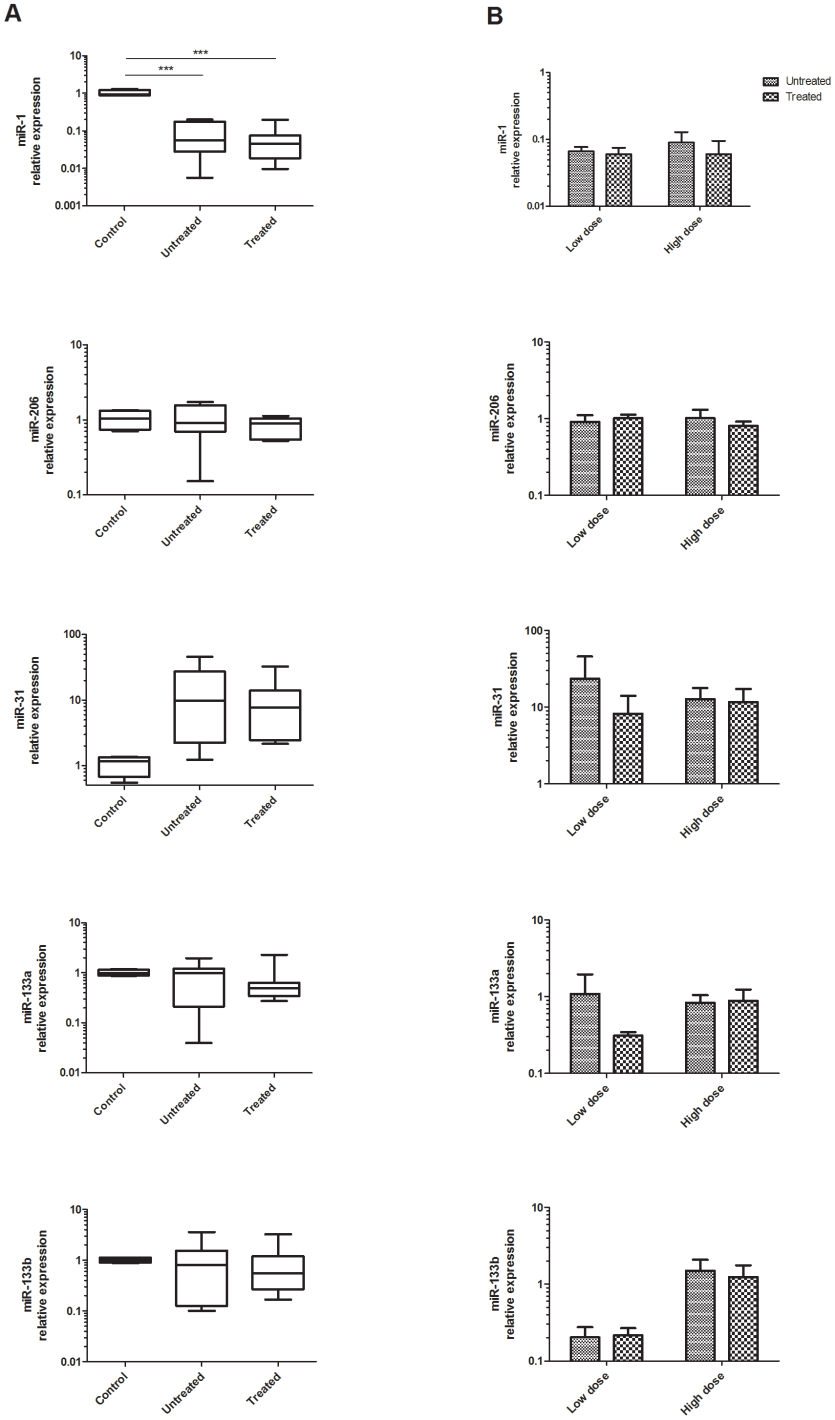
miRNAs as biomarkers for the outcome of therapeutic interventions: Intramuscular eteplirsen treatment trial. MiR-1, miR-206, miR-31, miR-133a and miR-133b expression in: A. untreated and treated muscle biopsy samples and controls; B. patients who received low dose and patients with high dose of eteplirsen. The data are presented in a logarithmic scale as relative fold change with respect to the control samples and normalized to U6 snRNA expression. P-values derived from two-tailed t-test are presented with *** and correspond to p<0.001.

MiR-1, miR-206, miR-31, miR-133a and miR-133b were quantified in untreated and treated muscle biopsy samples taken 3-4 weeks after the intramuscular administration of eteplirsen in all seven DMD patients. No difference in the expression of the dystromirs in the untreated compared to treated samples could be detected (Figure 7A).

To investigate whether the expression level of the dystromirs depended on the dose of eteplirsen and the amount of dystrophin restoration, we compared the untreated and treated samples in the two patients who received low dose (0.09 mg) and in the five patients who received high dose (0.9 mg). The analysed miRNAs did not showed a significant change in their expression level after treatment with the two different doses of eteplirsen (Figure 7B).

In addition, we analysed the expression of miR-1, miR-206, miR-31, miR-133a and miR-133b in muscle biopsies from four gender-matched controls and compared them to their expression in the DMD patients. The most differentially expressed miRNA in muscle samples was miR-1 which was decreased in DMD patients (untreated and treated muscles) compared to controls (p<0.001) (Figure 7A). MiR-31 was also found differentially expressed with mean fold increase of 15 in DMD patients and showed a trend towards significance (p = 0.057).

**Serum levels of miR-1, miR-206, miR-31, miR-133a and miR-133b in UCMD.** MiR-1, miR-206, miR-31, miR-133a and miR-133b were quantified in serum samples from patients with UCMD (n = 16) and the identified serum profile was compared to that detected in DMD patients and controls. In contrast to DMD patients, where there was an increase of the levels of miR-1, miR-206, miR133a and miR-133b in serum, none of the analysed five dystromirs showed any difference in their copy number in UCMD patients and their amounts were very similar to the levels found in controls (Figure 1).

Similarly to DMD, the analysis of the change of the levels in serum of the five miRNAs with age also showed decreasing amount of the miRNAs in UCMD patients but the profile was different between the two muscular dystrophies. Whereas in DMD we saw a considerable drop of the amount of miR-133a, miR-133b and miR-1 in patients after the age of 11 years, in UCMD we did not observe a substantial decrease in the level of these miRNAs in older patients (Figure 5B).

Further analysis of the amount of circulating miR-1, miR-133a, miR-133b, miR-206 and miR-31 in the serum of UCMD patients according to their ambulatory status and FVC score did not show a significant correlation between the level of the dystromirs and the analysed clinical variables.

## Discussion

We have performed quantification analysis of the muscle enriched miRNAs miR-1, miR-206, miR-31, miR-133a and miR-133b in a large cohort of serum samples from DMD, BMD and UCMD patients and in serum and muscle samples from DMD patients treated with exon skipping therapy. This study is the first to assess the application of these miRNAs as biomarkers for the disease progression in a large number of DMD patients and the first to investigate the possibility of utilising specific circulating miRNAs as non-invasive biomarkers for monitoring the outcome from exon skipping therapy in DMD patients. We also report for the first time the serum miRNA profile of the selected five dystromirs in UCMD, a muscular dystrophy with a different pathogenesis to DMD.

The first study that investigated circulating miRNAs as biomarkers for DMD found that miR-1, miR-133 and miR-206 were highly enriched in serum of DMD patients. The authors reported that the level of miRNAs correlated with the functional ability assessed using NSAA scores and higher levels of miRNAs corresponded to low ambulant activity [9]. Limitations of this study were the small number of patients analysed (n = 10) and the inclusion of only young, still ambulant, patients (<6 years).

The analysis that we performed according to the patients’ ambulation status and NSAA scores showed that ambulant patients have significantly higher amount of circulating miR-1, miR-206, miR-31, miR-133a and miR-133b compared to non-ambulant patients. However, the correlation analysis between NSAA scores and the level of the five dystromirs in the 26 ambulant patients did not show a significant correlation. The difference between the two studies is possibly due to the number of patients included in the NSAA correlation analysis. Our analysis is based on the data from 26 ambulant patients with available NSAA scores (age range from 4 to 13 years) whereas Cacchiarelli and colleagues studied 10 DMD patients with age range between 3 to 6 years of age. The larger number of patients and the broader age range most likely allowed us to establish a more extensive correlation between the miRNAs levels and the disease progression.

Contrary to the previous report [9] that increase in miRNA levels correlates with more severe muscle damage, our data show that milder DMD patients have higher levels of dystromirs compared to more severe DMD patients. All miRNAs were significantly increased in ambulant patients compared to non-ambulant patients. MiR-1, miR-31, miR-133a and miR-133b showed significantly higher levels in patients without the need of scoliosis surgery compared to patients with scoliosis surgery performed or recommended. Significantly higher levels of miR-1, miR-31 and miR-133b were also detected in patients on a daily steroid regimen, which has been shown to prolong ambulation and slow down the decline in DMD patients [25]. It is relevant to note that corticosteroid administration has an anabolic effect in DMD [26], hence it is likely that the higher doses of corticosteroids are associated with better muscle mass in treated boys and eventually higher levels of circulating miRNAs.

An important clinical parameter that provides information about the progression of respiratory muscle weakness is forced vital capacity value. A FVC <40-50% of predicted value significantly increases the risk of sleep disordered breathing and nocturnal hypoventilation and the need for non-invasive ventilation [27]. Linear regression analysis between the serum level of the five dystromirs and the FVC values in DMD patients revealed a significant positive correlation for miR-1 and miR-133b and the FVC value. Patients with low FVC values, indicating respiratory muscle weakness, have low levels of miR-1 and miR-133b and higher serum levels of the two miRNAs correspond to better lung function. These data further suggest that miR-1 and miR-133b might serve as non-invasive diagnostic markers for monitoring the progression of muscle weakness in DMD.

The analysis between the level of the five miRNAs in serum and the age of the DMD patients provided more information about the change of the amount of miRNAs over time. MiR-1, miR-133a and miR-133b showed a similar age pattern: higher and gradually decreasing level in patients between 4 to 10 years old and considerably lower levels of miRNAs in older patients (from 13 to 17 years of age). Based on the age profile of miR-1, miR-133a and miR-133b, we can speculate that the three dystromirs can be utilised as biomarkers for the remaining muscle mass in DMD. In the early stages of the disease the changes in the muscle pathology consist of fibre size variation and pronounced degeneration and regeneration processes. With the advance of the disease there is less regeneration activity, progressive loss of muscle fibres and their replacement by connective and adipose tissue. In the terminal stage of the disease, the muscles mainly consist of adipose tissue with residual muscle fibres [28]. We can speculate that in DMD the gradual loss of muscle fibres leads to gradual decrease of the amount of miRNAs released from the muscles and consequently a decrease in the amount of miRNAs in serum. Our data show higher amounts of miR-1, miR-133a and miR-133b in serum of DMD patients in the early stage of the disease (age 4 to 10 years) and decrease of these miRNAs with the progression of the disease where the low amount of circulating miRNAs might reflects the loss of muscle fibres and their replacement by connective and adipose tissue. However, more functional studies showing the relationship between the quantity of circulating miRNAs and the residual muscle mass in the dystrophic muscles are necessary.

When the expression of miR-1, miR-31, miR-206, miR-133a and miR133b was compared in muscle to serum from DMD patients, a difference in the profile of the dystromirs was seen which is consistent with previous reports in *mdx* mice [17], [20]. The greatest difference was found for miR-1, which had decreased expression in DMD muscle samples but was highly abundant in serum. The miRNAs that were upregulated in serum (miR-206, miR-133a and miR-133b) did not show a change in their expression in the analysed muscle samples. The differences in the profiles between muscle and serum detected in our data support the notion that the circulating miRNAs are not simply a result of “leaky” muscles, where the sarcolemmal damage causes spontaneous release of miRNAs from the damaged muscle into the blood. It has been proposed that miRNAs are actively secreted and released from the muscles as a result of a specific biological response to the dystrophic conditions [17].

Previous studies carried out in *mdx* mice showed elevated levels of circulating dystromirs, which were partly normalised to the wild type level after exon skipping therapy [9], [17]. Our analysis in DMD patients did not identify a significant difference when the levels of dystromirs were compared in serum samples before treatment and in post-treatment samples taken at week 12, when the last dose of eteplirsen was administered, despite an encouraging trend for normalisation of the miRNAs. Further analysis in the DMD patients according to the dose and response to eteplirsen did not reveal a conclusive result of the difference in the level of the studied miRNAs before and after treatment. We also analysed the same dystromirs in untreated and treated muscle samples in seven patients who received a single intramuscular infusion of eteplirsen. Similar to the results in serum samples, no difference in the expression levels of the dystromirs in untreated and treated muscle biopsies was detected and we could not detect a dose dependent change in the expression of the analysed miRNAs in patients who received either a low or high dose of eteplirsen [18].

The lack of a statistically significant difference between the untreated and treated samples could be due to a number of possibilities. One relates to the high variability that we observed in the levels of the analysed dystromirs in individual patients, which is in agreement with previous reports in *mdx* mice [17]. Due to the variability, a slight change in the levels of the dystromirs after therapeutic intervention will be difficult to identify when analysing small cohorts of patient samples, unless the therapeutic effect was overwhelming. The lack of statistically significant changes in the expression of miRNAs after exon skipping therapy may also be due to the small number of patients analysed. For example, only eight patients received higher dose of eteplirsen in the systemic clinical trial and a reliable assessment of the relationship between the outcome from exon skipping therapy and the change in the expression of dystromirs based on such numbers of patients represent a challenge. Another important point relates to the short duration of our study (12 weeks). Assessment of miRNAs in studies with longer duration, such as the recently reported eteplirsen 48 weeks trial, with doses of phosphorodiamidate morpholino oligomer up to 50 mg/kg/wk, could be more informative [29].

Indeed, a recent study in *mdx* mice using a morpholino antisense oligonucleotide, administered systemically at a dose equivalent to the one used in DMD clinical trials, showed that long-term administration increases dystrophin restoration [30]. The authors showed that the duration of the treatment was critical for the increase of dystrophin in skeletal muscle with the number of positive fibres and amount of dystrophin doubling after 50 weeks of treatment compared to 20 weeks. In our study the five dystromirs were analysed in serum samples taken after 12 weeks of eteplirsen treatment. A possible explanation for the lack of significant difference in the level of miRNAs in pre-treatment and post-treatment samples in patients recruited in our trial is that, although 12 weeks of treatment induced restoration of dystrophin expression in DMD patients [19], the 12-week time point might not be sufficient to assess response to therapy at the miRNA level, as the amount of dystrophin is still slowly increasing. The difference in the level of the circulating dystromirs in DMD patients with different severity and the higher amount of miRNAs in the patients with possibly more muscle mass that we detected in the current study suggest that miRNAs are valuable biomarkers in DMD. The quantification of miRNAs in serum after a more prolonged period of time might well be informative in treated boys and should be investigated in future clinical trials.

In the present study we also quantified miR-1, miR-206, miR-31, miR-133a and miR-133b in serum samples from Ullrich Congenital Muscular Dystrophy patients. In contrast to DMD, where we found an increase of miR-1, miR-206, miR-133a and miR-133b, none of the analysed dystromirs in UCMD showed dysregulated levels. In addition, there was a decrease in the amount of the serum miRNAs with age in UCMD patients, possibly due to the loss of muscle fibres with the progression of the disease, but the profile was different from that detected in DMD. The different pathological mechanism of DMD and UCMD might explain the differences in the levels of the analysed dystromirs in serum. In DMD the disrupted muscle membrane leads to gross leakage and elevated serum levels of creatine kinase (CK), followed by florid necrosis, regeneration and inflammation, while UCMD patients have normal or slightly elevated CK levels, and less signs of regeneration or inflammatory infiltration. This suggests that pathogenesis of the muscle fibre degeneration in UCMD is different from DMD [31], as also highlighted by recent data on mitochondrial damage, which is restricted to UCMD, and autophagy, more prominent in UCMD [32], [33], [34]. The dynamics of muscle regeneration are also very different between DMD and UCMD. The extracellular matrix in DMD is initially not disrupted, allowing satellite cell-mediated regeneration, whereas such regeneration does not occur in UCMD, possibly due to the effect of the lack of collagen VI on satellite cell function [35].

In a recent study, serum miRNAs were analysed in four mouse models for muscle pathologies: DMD (*mdx*), limb-girdle muscular dystrophy type 2D (*sgca*-null mice) and type 2C (*sgcg*-null mice) and Emery-Dreifuss muscular dystrophy (KI-*Lmna*) [20]. Upregulation of miR-1, miR-133a and miR-133b in serum was detected in the mouse models for DMD, limb-girdle muscular dystrophy type 2D (LGMD2D) and type 2C (LGMG2C) but the three miRNAs were slightly downregulated in the model for Emery-Dreifuss muscular dystrophy (EDMD). The results show that upregulation of miR-1, miR-133a and miR-133b in serum is not specific to DMD but is most likely associated with the pathological process occurring in the muscles. While a more pronounced dystrophic process, with muscle fibres wasting and cycles of degeneration and regeneration, is characteristic of DMD, LGMD2D and LGMG2C, in UCMD and in EDMD the dystrophic pathology is milder with lower level of regeneration and degeneration [20], [36]. Although more research in needed to show the relationship between the pathological process in dystrophic muscles and the release of specific miRNAs in the circulation, we can speculate that due to the different pathological mechanism in DMD and UCMD, different sets of miRNAs are expressed in muscle and in blood.

The urgent need for developing non-invasive biomarkers for the assessment of the muscle pathology in muscular dystrophy patients is defined by the fact that currently biochemical outcome studies of clinical trials need for a muscle biopsy, which is limited to a single time point, single muscle, is invasive and not desirable in children. MiRNAs represent an excellent class of blood-based biomarkers for DMD as they are present in a stable form in serum and plasma samples and their evaluation in blood-derived products represents a less invasive method compared to muscle biopsies. Although it remains difficult to conclude from our study whether circulating miR-1, miR-206, miR-31, miR-133a and miR-133b could be utilised as non-invasive biomarkers for monitoring the outcome from exon skipping therapeutic interventions, which will require longer studies than the one we have been involved with, we propose miR-1, miR-133a and miR-133b as reliable biomarkers for evaluating the disease progression and possibly monitoring the remaining muscle mass in DMD.

## Materials and Methods

### Study participants

**DMD, BMD, UCMD and healthy controls serum cohort.** The study was approved by the Berkshire Research Ethics Committee (REC reference 05/MRE12/32). Serum samples were supplied by the MRC CNMD Biobank London (REC reference number 06/Q0406/33) and were collected from individuals under a written informed consent of parents or legal guardians. All samples have been supplied to the project anonymised. Copies of the consent forms are kept with the Biobank in a locked cabinet and are also available in the patient's hospital notes at Great Ormond Street Hospital for Children and Hammersmith Hospital. Serum samples collection at the CMD comparative outcome measures study (COM) at the National Institutes of Health (NIH), Bethesda, MD, USA and Cincinnati Children’s Hospital Medical Centre (CCHMC), Cincinnati, OH, USA was approved by Cincinnati Children's Hospital Medical Center Investigational Review Board and all serum samples were collected from individuals under a written informed consent of parents or legal guardians.

The DMD and BMD patients were recruited for the study from the clinical population at Great Ormond Street Hospital, London. Sixteen DMD patients were non-ambulant with mean age of 14 years (age range between 10 and 17 years) and 28 DMD were ambulant with mean age of 8.2 years (age range 4 to 13 years). All BMD patients (n = 5) were ambulant with mean age of 13.6 (age range between 9 and 18 years).

Six serum samples from UCMD patients were collected at Great Ormond Street Hospital, London and ten samples were collected at CCHMC and the COM study at the NIH. Nine of the UCMD patients were non-ambulant (mean age 11.5, age range 5.9–16.8 years) and seven patients were ambulant (mean age 9.3 years, range 6.6–13 years).

Serum samples from fourteen healthy individuals were collected under a written informed consent: four serum samples were collected at Great Ormond Street Hospital, London and ten at Cincinnati Children’s Hospital Medical Centre. The mean age of the controls was 8.1 years and ranged between 2.9 and 15.9 years.

All serum samples are stored with consent in the MRC Centre for Neuromuscular Diseases Biobank, London (http://www.cnmd.ac.uk).

Additional information about the participants is provided in Table S1.

**Muscle biopsy samples from healthy controls.** Four skeletal muscle biopsies from gender-matched controls were obtained under written informed consent and stored in the MRC Centre for Neuromuscular Diseases Biobank, London.

**Muscle biopsies from DMD patients included in the intramuscular eteplirsen treatment trial.** The expression profiles of the selected dystromirs were analysed in skeletal muscle biopsies obtained under written informed consent from families and assent from patients included in the previously reported intramuscular eteplirsen treatment study [18] and stored in the MRC Centre for Neuromuscular Diseases Biobank, London. Ethical approval was obtained from the UK Gene Therapy Advisory Committee and local research ethics committees. The 7 patients studied were between 10 and 17 years and had a deletion that could be rescued by the skipping of exon 51 (Table 2). Eteplirsen was administrated into one extensor digitorum brevis muscle (EDB) and the contralateral muscle received an equivalent volume of normal saline. Bilateral EDB biopsies were obtained 3 to 4 weeks after treatment. The expression of the dystromirs was analysed in the biopsies from untreated and treated muscles from the two patients who received a low dose of 0.09mg of eteplirsen and from the five patients who received a high dose of 0.9mg of eteplirsen.

**Table 2:** Intramuscular eteplirsen exon skipping trial: Deletion and dose escalating scheme.

Patient ID	Dose eteplirsen	Deletion
P1	0.09 mg	del in intron 49 including the exon 50 acceptor splice site
P2	0.09 mg	del exon 50
P3	0.9 mg	del exon 48–50
P4	0.9 mg	del exon 48–50
P5	0.9 mg	del exon 45–50
P6	0.9 mg	del exon 45–50
P7	0.9 mg	del exon 49–50

**Serum samples from DMD patients participated in the systemic eteplirsen treatment trial.** Blood samples from the DMD patients who took part in a previously completed systemic clinical trial [19] were collected under written informed consent from parents and assent from children and stored in the MRC Centre for Neuromuscular Diseases Biobank, London. Ethical approval was obtained from the UK Gene Therapy Advisory Committee and local research ethics committees. The DMD patients were ambulant, aged 5–15 years with an out-of-frame deletion eligible for correction by skipping of exon 51 of the dystrophin gene. Eteplirsen was administrated in 12 weekly intravenous infusions. We analysed the level of the dystromirs in serum samples from DMD patients from cohort 3 (2 cases, 2 mg/kg), cohort 4 (2 cases, 4 mg/kg), cohort 5 (4 cases, 10 mg/kg) and cohort 6 (4 cases, 20 mg/kg) (Table 1). Analysis of the selected dystromirs was performed in serum samples obtained from blood taken >10 days prior to the first dose of study drug (baseline) and at week 12.

### RNA preparation and RT-PCR analysis

Serum samples were prepared from 1.5 ml to 4 ml of blood taken in BD vacutainer tubes. The blood sample was allowed to clot at room temperature for 30 minutes, followed by centrifugation at 2850g for 10 minutes. Serum supernatant was carefully collected, aliquoted in volumes of 200 µl in eppendorf tubes and stored at –80°C until use.

Total RNA was extracted from 200 µl serum samples using miRNeasy kit (QIAGEN) following the manufacturer’s protocol for liquid samples. For data normalization, we used a pool of artificial *Caenorhabditis elegans* miRs (*cel-miR-54, cel-miR-39, cel-miR-238*) added as a mixture of 25 fmol each in a 5 µl volume as previously described [37].

Total RNA from muscle biopsy samples was extracted using miRNeasy kit (QIAGEN) following the manufacturer’s protocol for tissue samples.

qRT-PCR was performed using TaqMan small RNA Assay (Applied Biosystems) following the manufacturer’s protocol. Briefly, a fixed volume of 5 µl of total RNA of a given sample was reverse transcribed using TaqMan MicroRNA Reverse Transcription kit. The PCR amplification of a volume of 1.33 µl cDNA was carried out using TaqMan Universal PCR master mix and miRNA-specific stem-loop primers (Applied BioSystems).

The copy number analysis of the level of miRs in serum was performed using standard curves prepared for each of the analysed miRNAs and a median normalization procedure with a normalization factor calculated using the Ct values obtained from the artificial spiked-in cel-miRs [37]. For the miRNA expression analysis in muscle samples we used the ΔΔCt method and U6 snRNA as endogenous control for data normalization.

### Statistical analysis

Comparisons between paired sample data were performed using a two-tailed Student's T test (two sample sets) and ANOVA for multiple sample data sets. Tests for linear relationships were carried out using linear regression. A p-value <0.05 was considered significant, allowing for multiple comparison correction, and analyses were carried out using R [38] and GraphPad (GraphPad Prism version 5 for Windows, GraphPad Software, La Jolla California USA, www.graphpad.com).

## Supporting Information

Figure S1 Serum level of dystromirs in healthy controls and DMD patients on different glucocorticoid treatment regimen. Level of miR-1, miR-206, miR-31, miR-133a and miR-133b in serum samples of healthy controls, DMD patients not on glucocorticoid treatment, on intermittent and on daily treatment regimen. The data are presented in a logarithmic scale as miRNA copy number per ml normalized to the spiked-in *C. elegance* miRNAs (*cel-miR-54, cel-miR-39, cel-miR-238)*. P-values derived from t-test are presented with *, ** and correspond to p <0.05 and p<0.01 respectively.
(TIF)

Table S1 The study cohort: DMD, BMD, UCMD and healthy controls. Presented are gender, age, ambulatory status, NSAA score, scoliosis surgery performed, FVC value and glucocorticoid regimen. NA: not available/not assessed.
(DOCX)
