# Supplementary figures and images for: Dystromirs as Serum Biomarkers for Monitoring the Disease Severity in Duchenne Muscular Dystrophy
Source: PLoS One. 2013 Nov 25;8(11):e80263. doi: 10.1371/journal.pone.0080263 (PMC3840009; doi:10.1371/journal.pone.0080263)

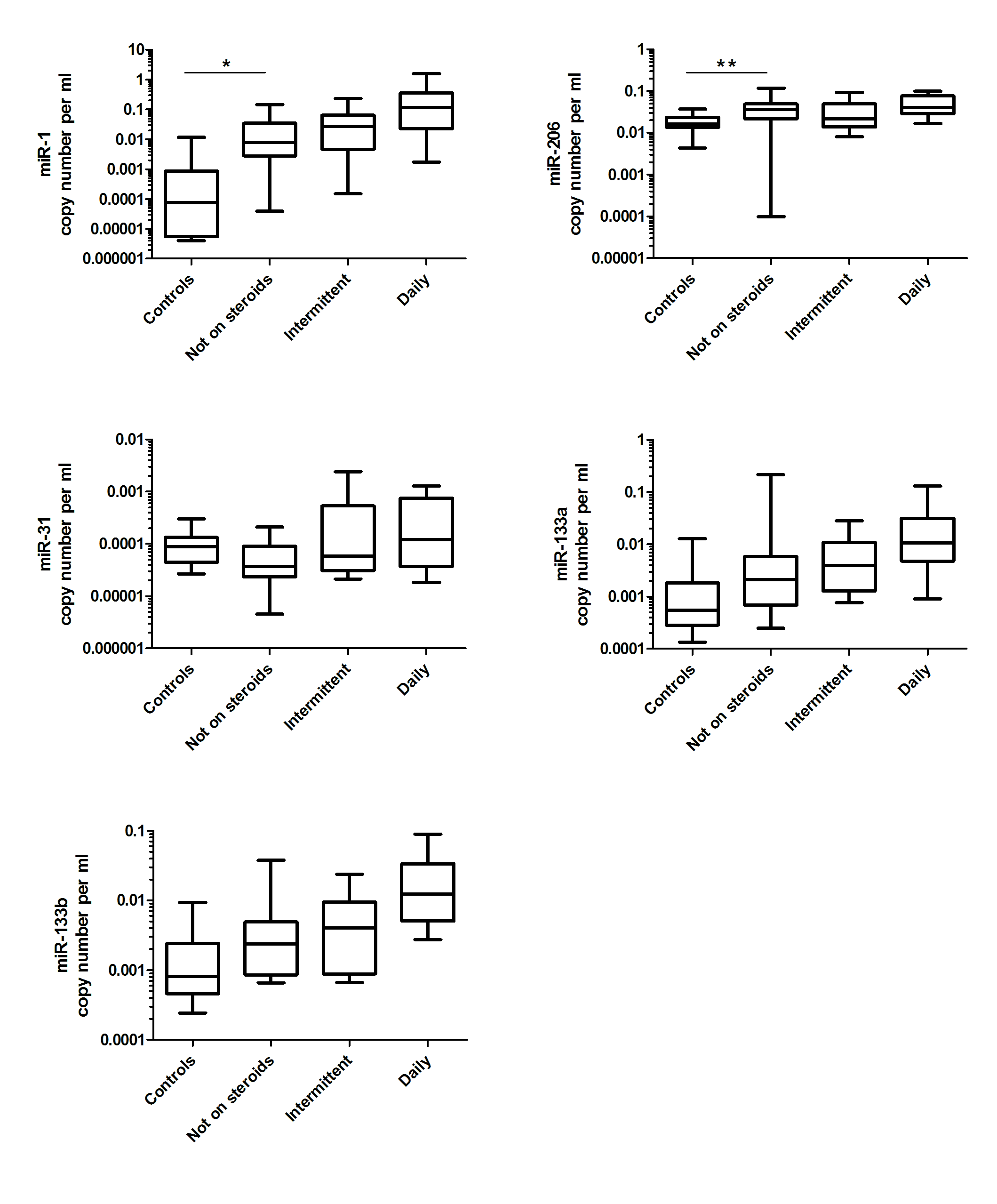

Supplement: Figure S1 — Serum level of dystromirs in healthy controls and DMD patients on different glucocorticoid treatment regimen. Level of miR-1, miR-206, miR-31, miR-133a and miR-133b in serum samples of healthy controls, DMD patients not on glucocorticoid treatment, on intermittent and on daily treatment regimen. The data are presented in a logarithmic scale as miRNA copy number per ml normalized to the spiked-in C. elegance miRNAs (cel-miR-54, cel-miR-39, cel-miR-238). P-values derived from t-test are presented with *, ** and correspond to p <0.05 and p<0.01 respectively. (TIF) [file pone.0080263.s001.tif]
