# Supplementary material for: Dystromirs as Serum Biomarkers for Monitoring the Disease Severity in Duchenne Muscular Dystrophy
Source: PLoS One. 2013 Nov 25;8(11):e80263. doi: 10.1371/journal.pone.0080263 (PMC3840009; doi:10.1371/journal.pone.0080263)
Supplement: Table S1 — The study cohort: DMD, BMD, UCMD and healthy controls. Presented are gender, age, ambulatory status, NSAA score, scoliosis surgery performed, FVC value and glucocorticoid regimen. NA: not available/not assessed. (DOCX) [file pone.0080263.s002.docx]

**Supporting Information**

**Table S1: The study cohort: DMD, BMD, UCMD and healthy controls.** Presented are the gender, age, ambulatory status, NSAA score, scoliosis surgery performed and FVC value. NA: not available/not assessed.
